# Land grabbing, CSR, and the (de)mobilization of resistance

**DOI:** 10.1007/s42597-024-00115-4

**Published:** 2024-03-18

**Authors:** Jan Sändig

**Affiliations:** University of Bayreuth, Bayreuth, Germany

**Keywords:** Large-scale agricultural investment, Land grabbing, Contentious politics, CSR, Cameroon, Großflächige Agrarinvestitionen, Landraub, Widerstand, Unternehmerische Sozialverantwortung, Kamerun

## Abstract

As large-scale agricultural investment has been rising, scholars have much investigated the factors that shape contestations against land grabbing. This literature, however, has hardly focused on the role of investing agricultural companies and their corporate social responsibility (CSR) practices so far. Vice versa, there is extensive research on the CSR-contention nexus for mining and other sectors, albeit with contested findings. To contribute to these debates, I apply the opportunities and threats framework from social movement studies to examine how CSR affects local and transnational contention. This is studied in the comparison of two major European agricultural companies that operate in Cameroon (and beyond). The analysis shows a demobilizing effect of timely and substantial CSR practices.

## Abstract (de)

Angesichts der Zunahme großflächiger Agrarinvestitionen hat sich die Forschung intensiv damit befasst, wie Auseinandersetzungen um Landraub (*land grabbing*) verlaufen. Diese Studien haben jedoch bisher kaum die Rolle der investierenden Agrarunternehmen und ihrer Praktiken der unternehmerischen Sozialverantwortung (CSR) fokussiert. Demgegenüber gibt es für den Bergbau und andere Sektoren zahlreiche Forschungsarbeiten zum Zusammenhang von CSR und Widerstand, wenngleich mit widerstreitenden Befunden. Als Beitrag zu diesen Debatten wende ich Theorien zu Gelegenheiten und Bedrohungen aus der Forschung zu sozialen Bewegungen an, um zu untersuchen, wie CSR lokale und transnationale Auseinandersetzungen beeinflusst. Dies betrachte ich im Vergleich zweier führender europäischer Agrarunternehmen, die in Kamerun (und anderen Ländern) tätig sind. Die Analyse zeigt eine demobilisierende Wirkung von frühzeitigen und substanziellen CSR-Praktiken.

## Introduction

Investment for large-scale plantations, mines, industry zones, ports, dams, conservation areas, and other projects has soared in the Global South lately (Hönke et al. 2024; Sändig and Schramm 2016). Proponents hail such investment for creating jobs, improving infrastructure, and increasing tax revenues. Yet, critics condemn them for land grabbing or “worldeating” (Dunlap and Jakobsen 2020). Indeed, possible development benefits are often undermined by the dispossession and displacement of local communities, by environmental damage, and by rent seeking practices (Lay et al. 2021). Unsurprisingly, thus, virtually all large-scale investment projects become contested in one way or another.^1^ However, the means of contestation have varied much (Sändig 2021): Some investment projects see rather limited actions (e.g. everyday resistance or localized collective action), whereas others turn into major contestations that reach into the national or even transnational arena.

The literature already provides various explanations why struggles against large-scale agricultural investment unfold differently (Hall et al. 2015; Sändig 2021). Key explanatory factors include the structures and resources of affected communities (Rutten et al. 2017), support from NGOs and political elites (Temper 2019), and repression (Dell’Angelo et al. 2021). Other studies point to framing processes (Prause 2019) and gender relations (Hennings 2019). While much attention has been placed on local communities, activists, and the political context, rather few studies have examined the investing companies (as similarly noted by Yang and He 2021). They started to show how agricultural companies use divide and rule tactics, consultation, and public legitimation efforts to prevent contentious actions (Gagné 2021; Vos et al. 2018).

In this context, I examine the still neglected question of how corporate social responsibility (CSR) practices impact contestation in the agricultural sector. In other areas, especially mining conflict research, this has already been widely studied. Business actors typically use CSR as management device in order to buy a “social license to operate” (Frederiksen 2018) and have an insurance against public critique (Godfrey et al. 2009). Yet, while some scholars find that CSR undercuts resistance (Bezzola et al. 2022; Maso 2024), others argue that CSR inadvertently incentivizes contentious actions (Haslam and Godfrid 2023; King and McDonnell 2015). What further complicates the analysis is conceptual fluidity and causal complexity (Crane et al. 2014). For instance, structural corporate traits like business size, sector, and geographic origin also shape contestations (Bartley and Child 2014; King 2008).

The paper pursues two goals: it seeks to elucidate how CSR practices impact contention against large-scale investment in the agricultural sector, while also addressing the competing findings within the broader study of the CSR-contention nexus. For this purpose, I draw on the opportunities and threats framework from social movement studies (Tarrow 2011, p. 160–175). This already proved relevant to explain struggles against agricultural companies (Gingembre 2015; Famerée 2016). From this perspective, I examine how CSR practices may shape the grievances, threats, and opportunities underlying contentious actions at multiple levels.

Empirically, I turn to the Cameroonian branches of two major European agricultural companies: the *Société Financière des Caoutchoucs* (SOCFIN) and the *Société d’Organisation, de Management et de Développement des Industries Alimentaires et Agricoles *(SOMDIAA). These cases were selected because these large European agricultural companies are highly exposed to the CSR agenda and share structural similarities, which make them likely targets of local and transnational resistance. Yet, these companies have been differently contested, which leads me to ask if differences of CSR endorsement matter for the contention. The analysis draws on interviews mainly from Cameroon and primary documents, like activist magazines, company publications, social media, and news sources.

The findings align with studies that show a demobilizing effect of CSR for contestation (Haslam 2021; Maso 2024), while specifying that timing and the extent of CSR matters. I find that CSR practices can indeed mitigate grievances, prevent threats to local livelihoods, and enable dialogue as alternative opportunity for redress. Yet, if CSR is merely rhetoric, if these practices are non-substantial or become discontinued, and especially if corporate practices obviously contravene the CSR expectations, this provokes contestation both on the ground and transnationally. Moreover, CSR does not curb contestation if undertaken only belatedly in response to an already highly mobilized movement.

## Theories—Large-scale investment projects, CSR, and contentious actions

### State of the research

The literature details the varied positions that local, national, and transnational actors take vis-à-vis large-scale plantation projects. Faced with such investments, actors often become divided into proponents and opponents (Borras and Franco 2013). Among local communities, some groups usually promote the investment and seek employment, others tolerate the company’s presence or migrate, and still others pursue contentious actions (Hall et al. 2015). While contestations seem to occur in any case, the means, extent, and scales can vary: Affected people often use everyday resistance (Scott 1985), e.g. by trespassing company grounds, ridiculing investors, stealing from the company, or destroying its property. More organized resistance comprises collective action (e.g. marches, road blockades, land occupation) and rights-based means, such as petitioning and litigation (Dell’Angelo et al. 2021). This may surpass the local arena, e.g. when civil society groups file complaints to investment grievance mechanisms, appeal to state actors, or publicly shame companies over rights violations. Hence, the question usually is not whether contestations occur but *why they unfold differently*.

Emerging from political ecology, critical agrarian studies, and social movement research, the literature already highlights various explanatory factors (Hall et al. 2015). Whereas everyday resistance is individual action that requires little else than grievances, collective contention also needs local solidarity, resource mobilization, political opportunities, and apt framings (Sändig 2021). Such actions, thus, occur if communities are rather united, have support from chiefs and other local elites, and have (or can mobilize) resources like money, time, and land titles (Gingembre 2015; Rutten et al. 2017; Schoenberger 2017). Support from outside political allies, NGOs, and broader activist networks provides important means and leverage to broaden contestation beyond the local arena (Gerber 2011; Temper 2019). Especially in repressive settings, this usually entails rights-based struggles (Kenney-Lazar et al. 2018; Schramm 2020). Relatedly, activists use framing strategies, such as appropriating state discourses, to denounce injustices and shield themselves from repression (Prause 2019).

Vice versa, contestation against agricultural investment is constrained by resource scarcity, repression, and community fragmentation. Such investment projects in the Global South typically involve great power asymmetry between resource-scarce rural communities and well-endowed investors, supported by the state. In this setting, local communities are easily overwhelmed and often face repression through intimidation, criminalization, and the outright killing of land rights activists (Dell’Angelo et al. 2021; Lund 2018). The companies also usually forge close ties with local leaders and state authorities and frame the investment in line with the state’s development agenda to prevent opposition (Alonso-Fradejas 2015; Gagné 2021). As a result, communities often fragment and engage in infighting, which undermines collective resistance. Yet, even under such unfavorable circumstances, local actors may find means to organize resistance, often with the help of civil society and state allies (Kenney-Lazar 2018; Sändig and Schramm 2023).

There has been surprisingly little focus on the actions and strategies of agricultural companies within this literature so far. Scholars in the field often come from an activist background and consider agricultural companies as rather uniform actors or mere expressions of global capital. Being skeptical of state and corporate commitments to responsible land governance and human rights (Borras and Franco 2010; Clapp 2017), scholars tend to advocate agrarian reform and smallholder-based agriculture (McKeon 2015). Nevertheless, as noted in a recent literature review, “investors are not a monolithic block; they are far more diverse than current researchers claim in terms of behavioural differences and motivations for land deals” (Yang and He 2021, p. 14).

Some studies have already examined more closely how agricultural companies operate and shape contestation. These studies illustrate a certain corporate repertoire of control (Gagné 2021), which comprises ambitious development promises, community consultation, cooptation, sidelining of opponents, and public relations efforts along national development goals. Apart from cases of blunt, violent land grabbing (Lund 2018), companies more often exploit the local lack of information for flawed consultation processes in which local communities have no effective decision-making power (Schramm 2020; Vos et al. 2018). Other studies point to companies’ political alliances and the distribution of patronage for curtailing contestation (Hennings 2018). Amid the limited research that explicitly engages with CSR practices, some suggest there is a business case for CSR as it helps agricultural investors to evade costly conflicts with local communities (Feyertag and Bowie 2021).

Beyond agricultural investment, there has been significantly more research specifically on the role of CSR for contention. However, this has been challenging to study due to conceptual vagueness. CSR encapsulates the normative idea that companies should be good citizens through high social, environmental, and corporate governance standards (Crane et al. 2014). In practice, the CSR repertoire ranges from some charity to full social entrepreneurship (Schneider 2015). For large-scale land-based investments, CSR typically encompasses social and environmental impact assessments, community consultation, the creation of grievance mechanisms, environmental management systems, and increased business transparency (Feyertag and Bowie 2021). Particularly prominent has been CSR spending for local development through basic infrastructure construction (e.g. wells, schools, health posts, roads) and capacity-building.

While investors typically expect that CSR helps them to shield their business from resistance (Frederiksen 2018), research on mining and other sectors suggests ambiguous effects for contention (Conde and Le Billon 2017). On the one hand, scholars find demobilizing effects of CSR when such programs effectively lower local perceptions of uncertainty and risk, provide meaningful dialogue, and distribute tangible resources (Haslam 2021; Feyertag and Bowie 2021). These CSR efforts are usually part of broader corporate security practices, which also include means like fencing and clientelism that aim at obstructing local resistance (Hönke 2018).

On the other hand, CSR practices can inadvertently arouse contestation. As CSR policies are often rhetorical devices and empty shells, they can incentivize communities to engage in contentious actions to push for (further) CSR implementation (Haslam and Godfrid 2023). CSR efforts can also entail distributional struggles and motivate resistance for improving company-provided dialogue platforms (Anguelovski 2011; Bezzola 2022). Furthermore, research on Western corporations of various size showed that activists prefer targeting companies that engage in CSR actions, because CSR signals reputational vulnerability, which makes concessions more likely (King and McDonnell 2015; Graafland 2018). Hence, there are competing findings within the broader literature on how CSR affects contestation.

### Opportunities and threats framework

The theoretical framework for the empirical analysis integrates these partly competing findings on CSR effects for contestation into the well-established opportunities and threats perspective from social movement studies (McAdam et al. 2001). This perspective assumes that activism requires both a motivation, due to grievances and threats, as well as action opportunities. Whereas grievances (i.e., anger and frustration about an issue) were long seen as key driver of protest, recent studies show that people particularly take action if they consider their livelihoods threatened (Almeida 2019). Yet, actions also require circumstances that make them feasible and promising—hence political opportunities. These can emerge from civil and political rights, access to political institutions, and the formation of political alliances (Kriesi 2004). Over the course of contestation, opportunities and threats can also shift, as protesters, their target, and the audience interact (McAdam et al. 2001).

Drawing on this framework, I carve out three dimensions of how CSR may shape resistance against agricultural investment. These dimensions are partly interrelated and dynamic.

The first dimension looks at how companies, especially through CSR, shape local grievances, threats, and potential benefits. Evidently, the land acquisition itself has the largest local impact and often is the main source of contention (Gerber 2011). Land acquisition comes in many shades. The worst is outright land grabbing where people are (violently) dispossessed from agricultural areas, forests, and sacred sites, which are key to their livelihoods. But there can also be agricultural investment without land enclosure, e.g. through smallholder schemes, which tend to become much less contested. Apart from land acquisition, contestation can arise over other negative impacts, such as repression, pollution, and other environmental damage (Dell’Angelo et al. 2017).

CSR is set out to limit detrimental impacts and increase benefits for local stakeholders. Common CSR standards require agricultural companies to respect informal tenure rights, ensure meaningful participation of affected groups, provide remedy and compensation for losses, and contribute to rural development and food security (FAO 2014).^2^ These measures seek to prevent land grabbing, reduce negative local impacts, and increase potential benefits. If effectively implemented, this would lower contestation by reducing grievances and providing tangible benefits (Haslam 2021). Then again, CSR projects often fail to mitigate negative consequences of the investment, deepen local frustrations, and entail distributional conflicts over benefits, thus arousing further resistance (Warnaars 2012; Bezzola 2022).

The second dimension focuses on local action opportunities. As mentioned, companies regularly seek to curtail resistance through fencing, clientelistic relations with local, regional, and national political authorities, and CSR practices (Hönke 2018; Bechtum 2024). Hence, they have significant repression and cooptation capacities, which further constrain the already limited action opportunities of most rural communities in the Global South.

Community consultation, dialogue efforts, and grievances mechanisms are key CSR means that affect the opportunities for contentious actions at the plantation level. CSR standards call upon agricultural companies to actively engage in meaningful dialogue with affected local constituents and particularly with vulnerable groups, prior to taking decisions (FAO 2014, p. 17). More inclusive forms of dialogue can give communities a say in company decisions, thereby providing opportunities for raising claims by means other than contentious actions (Feyertag and Bowie 2021; Haslam 2021). Yet, in reality, companies have often provided insufficient information, allowed limited participation, and sought after-the-fact approval (Perreault 2015). Flawed consultation can both hinder and motivate contentious actions (Anguelovski 2011; Vos et al. 2018).

Thirdly, given the focus on foreign investment, opportunities and threats also need to be considered at the transnational level. International NGOs (INGOs) are key actors of contestation in this arena (Temper 2019). Given resource constraints, they need to select out of a large pool of potential campaigning cases (Bob 2005). For them, companies that have certain structural properties make attractive targets. INGOs tend to engage particularly on large, Western companies that have brands to defend and that are headquartered in the activists’ home countries (Bartley and Child 2014; Hatte and Koenig 2020; Sändig and Hönke 2024). This allows INGOs to rally consumer power through “naming-and-shaming”, lobby politicians, and pursue legal avenues against the company.

CSR practices tend to open opportunities for transnational activism. Through CSR endorsement, foreign investors increase their reputational vulnerability and public visibility, disclose more information on their business operations, and often endorse responsible investment initiatives, which come with extra complaint mechanisms and public attention (King and McDonnell 2015; Graafland 2018). CSR-committed companies are usually also more responsive (McDonnell et al. 2015), which makes them attractive for further activist challenges.

In contrast, threats for transnational activism have hardly been studied so far. Research on repression has mainly focused on threats at the investment site, especially within the Global South (Dell’Angelo et al. 2021). As the case of Global Witness demonstrates, however, Northern-based activists can also experience repression for uncovering corporate actions. This may have diverse effects, both discouraging and amplifying contestation.

## Cases and methodology

To examine how CSR practices shape contestation, I selected cases of agricultural companies that meet certain structural conditions (like large size, Western origin, brand vulnerability), which give them high exposure to the CSR agenda and high activist attention. Yet, the selected cases differ in the extent and scale of contestation, which allows assessment of the role of corporate practices, especially of CSR, for contention.

### Case selection and background

The empirical analysis compares the Cameroonian branches of SOCFIN and SOMDIAA. These are the *Société Camerounaise de Palmeraies *(SOCAPALM) and the *Société Sucrière du Cameroun *(SOSUCAM), respectively.

Considering the relevance of company size for contention (King 2008), I focus on large companies that have significant local impact and international visibility.^3^ SOCAPALM is Cameroon’s largest palm oil producer, belonging to SOCFIN since 2000. The company operates six sites in Cameroon’s coastal region, north and south of Douala. SOCAPALM holds 58,500 ha of land concession with 35,000 ha planted, mostly for palm oil. Since 2014, the adjacent rubber plantation SAFACAM (15,500 ha concession) also belongs to SOCFIN. Overall, SOCFIN has 400,000 ha of land concessions, operating 190,000 ha of palm oil and rubber plantations in ten countries of Africa and South-East Asia.

SOSUCAM and SOMDIAA are major agricultural companies, too. SOSUCAM is located at Mbandjock and Nkoteng in Cameroon’s Center region. Its plantation covers 18,700 ha of sugar cane on a 25,000 ha concession area. It is the largest plantation of SOMDIAA, which operates 11 branches in eight African countries for sugar, flour, corn, and animal food production. This includes 80,000 ha concession area with 56,000 ha of sugar cane plantations in six countries of Central and Western Africa. Albeit smaller than SOCFIN, is still one of Europe’s largest agricultural investors in Africa.

Such large Western-based companies likely become monitored by INGOs (Hatte and Koenig 2020). As a multinational group, SOCFIN is based in Switzerland, France, Luxembourg, and Belgium. Although it is a publicly listed company, SOCFIN essentially belongs to the Belgian investor Hubert Fabri (54% of the shares) and the French Vincent Bolloré (39%).^4^ SOMDIAA, in turn, is a French company. Founded by the Vilgrain family in 1947, SOMDIAA has long been family-run, last by Alexandre Vilgrain (CEO from 1995 to 2022). But since 2011, it is part of the beverage producing Castel Group.

While INGOs prefer targeting companies that have brands to defend (Bartley and Child 2014), SOCFIN and SOMDIAA themselves have no internationally-known brands. Yet, both are connected to brands, which make them indirectly vulnerable to activist challenges. SOCFIN’s brand vulnerability results from its major shareholder Vincent Bolloré. The French billionaire is CEO of the Bolloré Group, one of the largest media and logistics companies in France. He is a brand by himself, and the Bolloré Group has stakes in media houses including Vivendi, Havas, and the Universal Music Group. SOMIDAA, in turn, is part of the Castel Group, which is one of the largest wine producers worldwide, a major brewer in Africa, and supplier of The Coca Cola Company. Hence, there are brands that can be invoked for transnational activism in both cases.

Looking at the Cameroonian branches, further similarities of corporate structures and historical developments allow comparison. Unlike most cases discussed within the land grabbing debate, these are mainly old plantations rather than newly developed ones. SOSUCAM was founded as a private company in 1964 and SOCAPALM as a state-owned enterprise in 1968. In both cases, large concession areas were acquired through presidential decrees that expropriated local communities during the 1970s and 1980s. SOSUCAM also took over the adjacent state-run Cameroon Sugar Company (CAMSUCO) in 1998. In 2006, it leased another 12,000 ha of land from the Cameroonian state for extensions.

Similarities also exist in the ownership structure, production model, and political context. In both companies, the Cameroonian state and domestic investors hold major stakes, owning 33% of SOCAPALM and 27% of SOSUCAM. Both companies rely heavily on harsh manual labor with a similarly sized workforce (roughly 5000–6000 employees each) and use of subcontractors. There is seasonal employment at SOSUCAM, however. Moreover, the political, legal, and socio-economic context within Cameroon is similarly characterized by rural poverty, insecure land tenure, and ethnic polarization (Kenfack et al. 2016).

### Charting the contestations

Despite these numerous structural similarities, the companies have been differently contested. SOCFIN has become one of the most prominent cases of transnational activist campaigning against land grabbing, with SOCAPALM being a key site. The contestation started in 2009 when land grabbing rose to international prominence. Back then, local activists, Cameroonian NGOs, and their international partners documented grievances. The group filed a complaint with the French National Contact Point (NCP) of the Organization for Economic Co-operation and Development (OECD) in 2010. Assisted by a French NGO, local activists soon created a grassroots network, the *Synergie Nationale des Paysans et Riverains du Cameroun* (SYNAPARCAM). In numerous instances, the activists blocked roads, marched in protest, and petitioned the company and state authorities. There has also been everyday resistance as people collect palm fruits from the concession area for private palm oil production.

These contestations have been part of—and have nurtured—the broader protest campaign against SOCFIN. Similar local actions have occurred at SOCFIN branches in Cambodia, Liberia, and Sierra Leone. In parallel, European activists have protested at the headquarters of SOCFIN, the Bolloré Group, and lending banks. Recently, NGOs also filed formal complaints to the NCP and the International Finance Corporation (IFC) over SOCFIN’s Liberian plantation, and they brought SOCAPALM to court at Paris. This major protest campaign has been ongoing for more than a decade.

SOSUCAM and SOMDIAA have been far less contested in general, but three stages can be distinguished. In the first stage, around 2010, SOSUCAM briefly encountered both local and transnational resistance. At the time, the company sought to plant on newly acquired land, yet communities blocked the construction and rallied in protest. ActionAid France petitioned the company, calling the situation “symbolic of the worldwide phenomenon of land grabbing” (Peuples Solidaires 2010). ActionAid France and Friends of the Earth also awarded SOMDIAA with the mocking Pinocchio Prize.

In a second stage, from 2011 to 2020, only scattered, small-scale, and localized contestations occurred at SOSUCAM and other SOMDIAA branches. Interviewees recounted few and little organized attempts by community members to block roads and instances of arson at SOSUCAM. At SOMDIAA’s Ivoirian branch, local chiefs petitioned the company, marched in protest, and let their cattle graze on company grounds (Partner Africa 2017a, pp. 28–30).

In the third stage, starting in late 2020, the company has encountered increased local and transnational contention. In late 2020, the Yaoundé-based *Centre d’Action pour la Vie et pour la Terre* (CAVT) filed a complaint against SOSUCAM with the French NCP. Concomitantly, the *Réseau pour l’Action collective transnationale* (ReAct Transnational) denounced the Castel Group, mostly for poor working conditions at SOSUCAM and other branches (ReAct Transnational 2022). An investigative group also reported that SOMDIAA may have funded militias who committed atrocities in the Central African Republic (CAR, see The Sentry 2021). Hence, while the level of contention is still lower than in the SOCFIN case, there has been a notable surge lately and an important variation over time at SOMDIAA.

### Data collection, positionality, and caveats

Information was collected mainly from 52 interviews with a total of 58 respondents. Most interviews were conducted during field research in Cameroon in November/December 2017. The interviewees comprise people from local communities (young people, women, elderly, traditional leaders, other local elites) around the plantations, grassroots activists, NGO staff, state representatives, and low-level company staff.^5^ Follow-up online interviews with INGOs served to explore the transnational angle. For triangulating the interview findings and documenting corporate practices and protest events (also beyond Cameroon), I relied on activist publications, online newspapers, social media, and company reports. The time frame of the analysis starts in 2010 when land grabbing hit the news and contestation took off at both companies.

Challenges arose considering that company-community conflicts are sensitive and that access is often limited for outside, Western researchers. To address this, I worked with the Yaoundé-based NGO *Réseau de Lutte contre la Faim* (RELUFA) and two local civil society figures. They helped to identify interviewees, create a setting where people felt safe and confident to speak, and ensured that cultural expectations were met. These well-connected civil society figures were also great sources of knowledge on the localities, their history, and the political context. Their support provided important access, particularly to the views of local communities, yet a potential bias is that interviewees may have overemphasized negative views of the companies. Moreover, access to political authorities and the companies remained restricted.^6^ Informal and off-the-record talks with low-level company staff were possible but document analysis was much used for contextualization.

There are further caveats. Given the focus on Cameroon and the European headquarters, only scarce evidence was collected and included on the companies’ other branches. Worker struggles were disregarded for space constraints, even though they also contributed to some contestation and INGO involvement. A closer look at the role of the Cameroonian state, as stakeholder of both companies, would also have been needed. Yet, I lacked the access to key actors to disentangle the relationship. The closer analysis of potential learning processes between the examined cases is another relevant aspect for further research. Finally, a common issue with small‑N comparison is external validity. There are certainly country and case particularities, such as the fact that these are mainly old plantations. However, Cameroon also shares features with other Southern countries, especially regarding the autocratic and highly personalized political regime, rural poverty, and ethnic polarization. Moreover, land acquisition processes that activists denounced as land grabbing have occurred here, too. There is, thus, both some external validity and a general relevance for current land grabbing debates.

## Comparative analysis

### Local grievances and threats: Land acquisition, control, and community projects

The operations of both companies have created intense local grievances, which are common for such large-scale plantations. A major issue in both cases is the shortage of local employment. As an interviewee at SOSUCAM expressed: “Since we gave so much land, a proportion of the jobs should be reserved for us. That means labor force, supervisors, managers.”^7^ Local people often complained about the hiring practices, suspecting ethnic bias. Indeed, the workforce of SOSUCAM comprises a large share of migrant workers from Cameroon’s Far North, and SOCAPALM widely recruits Anglophone Cameroonians.

Moreover, people expect the companies to provide more development benefits. As a young interviewee at SOCAPALM argued: “The hospitals, schools, and electricity that we asked for, they did nothing. They hire very few young people from the village, only three or four. They only exploit us. (…) They built a football field, but that is nothing.”^8^ Similarly, some local respondents called SOSUCAM a “machine” that neglects people’s basic needs. Furthermore, environmental and health issues are regularly raised. This mostly concerns air and water pollution as well as noise near the factories of Mbongo (SOCAPALM), Mbandjock, and Nkoteng (both SOSUCAM). At SOSUCAM, there are also complaints that pesticide spraying destroys local crops and damages roofs.

The companies’ practices have differed in key regards, however, which shaped local perceptions of threat and thereby impacted resistance. SOCAPALM’s concession was created through a series of presidential decrees that expropriated local communities, mostly during the 1970s. As a result, arable land has already been scarce in many areas, most importantly Mbonjo village, which is surrounded by the plantation. In this context, SOCAPALM’s “belligerent” practices of land governance have provoked resistance (SNJP 2016). Interviewees reported recent attempts by the company to clear peripheral areas within the concession for further plantation development. This poses major threats to local livelihoods, which depend on these areas: “And SOCAPALM is even in the process of taking over the periphery. How are we going to live? (...) On the periphery, we plant macabo, manioc, banana.”^9^ Such encroachment and the shortage of communal land infringes the terms of the land lease agreement regarding a buffer zone for local agriculture (SNJP 2016, p. 21).

SOCAPALM also retained control of up to 20,000 ha, which the company agreed to retrocede (give back) to local communities in 2005 (SNJP 2016, p. 25). The retrocession would alleviate land scarcity, yet only marginal areas have been returned to local communities. Respondents argued that company or state elites block the retrocession, which affects local livelihoods:

We learned that the state had retroceded to us 240 hectares that we had never known until today. The company has taken everything, where do we grow? They have to give it back but it has never been done. It is a company that does not take care of the local residents.^10^

Moreover, SOCAPALM has repressed communal actions. Local people have regularly engaged in everyday resistance through collecting palm fruits from the plantation for private palm oil pressing, which also serves to secure livelihoods. Yet, company security operators seek to repress this by means reportedly including extortion, beatings, and rape.^11^ This led to an escalatory spiral, in which local people, who see their livelihoods threatened, engage in everyday resistance, which then leads to harsher repression.

Nevertheless, SOCAPALM has framed its corporate practices as respecting CSR. The company endorsed an ethical charter in 2009 and has released CSR reports since 2013. It constructed houses, roads, and wells, provided medical care, and paid teachers’ salaries (SOCAPALM 2014). However, these efforts have been directed more at the workforce than the local communities. Local respondents uniformly declared that they gain nothing substantial from the company. The CSR actions also clearly fail to address the key contested issues, particularly land access and repression.

At SOCFIN’s other branches, similar practices of land governance and superficial CSR can be observed. Complaints about the grabbing of vital communal land with insufficient consultation and poor compensation arose in Sierra Leone, Liberia, and Cambodia (Bread for All 2019; FIDH 2011). The Sierra Leonean case stands out, considering heavy livelihood impacts and severe repression against a grassroots activist network (Sändig and Schramm 2023). Here, too, SOCFIN provided some compensation by constructing wells, toilets, and streetlights, yet “these CSR projects do not seem to be able to replace proper grievance mechanisms and transparent negotiations” (Schramm 2020, p. 166). As the CSR practices have been substandard and as the company has threatened local livelihoods, it pushed people into contentious actions in Cameroon and beyond.

Early on, SOSUCAM pursued similar non-consultative practices of land acquisition. In 2010, the company sought to clear communal lands for extending the plantation by 12,000 ha. This was based on a land lease agreement, signed in 2006 without prior and meaningful consultation of local communities. The latter then blocked the land transformation and ActionAid France denounced SOMDIAA for land grabbing (Peuples Solidaires 2010).

In response to this outcry and the rising international attention on land grabbing, the company endorsed CSR practices and declared evading land grabs its goal. SOSUCAM subsequently pursued a series of community meetings to ensure that the extensions have no impacts on local land needs and livelihoods. As an activist recounted:

In [name of village withheld], during the extensions, they realized that the population had no more land to cultivate, because the company had taken everything. We debated with the company to claim this. In the end, the company gave back, I think, 100 ha to the people for crops.^12^

This has been largely effective, as interviewees around the plantation generally recognized that they have sufficient agricultural areas left. The communities also do not reclaim land from the company but rather ask for more compensation through jobs, infrastructure development, and higher land lease payments. Moreover, SOSUCAM has not used private and state security actors for repressing local dissent. There have been no such escalatory spirals over everyday resistance, as observed at SOCAPALM. But this has also been easier to evade here since sugar cane is less “lootable” than palm fruits.

As part of its CSR efforts, SOMDIAA created foundations at all plantations between 2010 and 2012. The foundations have assisted local associations and funded small-scale projects for education, income generation, and social services (SOMDIAA 2017). SOSUCAM’s foundation provided wells, school equipment, and other local assistance, as part of a five-year environmental and social plan from 2012 on. In a simple but popular move, SOSUCAM organized a twice daily bus service, which is free of charge and connects remote villages to urban places. This increases local commercial activities and improves income generation. As a result, most interviewees at SOSUCAM recognized some benefit from the company.

SOMDIAA acted similarly at its other branches. The company has been at some advantage over SOCFIN considering its lower pace of plantation expansion. Therefore, conflicts at the SOMDIAA sites in the Côte d’Ivoire (CIV), Gabon, and the Republic of the Congo have centered more on the demands for local employment, construction or rehabilitation of local infrastructure (roads, electricity, schools, clinics), assistance for community-based organizations, local subcontracting, and inclusion through smallholder schemes (Partner Africa 2017c, pp. 19–20, 2017b, a, p. 19). Where SOMDIAA acquired new land, local needs were respected more than in the case of SOCFIN. For instance, the company refrained from displacing a village for extending the planted area within the Congolese concession (Partner Africa 2017c, p. 19).

Overall, there is a clear link between SOCFIN’s aggressive approach to land acquisition, use of repression and its superficial CSR practices and the heated contestation at its multiple branches. In contrast, SOMDIAA’s actions have aligned more with common CSR standards, at least for most of the 2010s. This has contributed to lowering community grievances and evading land grabbing, thereby limiting local contention at SOSUCAM and other branches.

### Local opportunities: Dialogue and NGO involvement

As common for such investment sites in the Global South, the opportunities for local resistance have been limited in both cases. The affected communities have lived in rural poverty, lacking crucial resources, such as money, formal land titles, knowledge of legal procedures, and political connections. Although there have hardly been community divides that could undermine contention, the shortage of political support has been an obstacle. State actors from the local level via regional authorities to high-level institutions have sided with the companies. This is unsurprising, considering the state’s stakes in both companies and their relevance for national revenue. While some village chiefs have been supportive of contentious actions, not all of them have. For instance, in the extension areas SOSUCAM has distributed 20% of the annual lease rent to chiefs. This only amounts to a few thousand dollars per village and year, but it still disincentivizes contentious actions.^13^ Opportunities for legal recourse are also limited under Cameroon’s autocratic regime. The costs of litigation are prohibitive for most people, courts are slow, and the judiciary lacks independence from the state. Additionally, SOCAPALM’s multi-site structure imposes high logistical costs for mobilization.

Facing these constraints, interviewees often expressed that they felt powerless vis-à-vis the companies: “We are afraid of opposition, we are afraid. There is politics. Do we have the power? If they have the money and we do not, we cannot challenge them.”^14^ Similar constraints apply to the other branches: Poverty, insecure land tenure, and autocratic rule similarly prevail at SOCFIN’s plantations in Sierra Leone, Cambodia, and Indonesia as well as at SOMDIAA’s subsidiaries in Gabon, the CAR, and the Republic of the Congo.

Through their approaches to community dialogue, the companies have further shaped the local action opportunities. SOCAPALM and SOCFIN have evaded and dismissed dialogue, which has fueled contestation. From early on, SOCAPALM took a non-cooperative stance. This led local activists and Cameroonian NGOs, together with NGOs from France and Germany, to file the NCP complaint in 2010. The Bolloré Group, as major shareholder of SOCFIN, only hesitantly participated in the NCP mediation. Eventually, in 2013, it accepted a roadmap for improving dialogue with the communities at SOCAPALM, addressing land-related conflict, and contributing more to local development (PCN France 2014). Yet, the company refrained from implementing the agreement (PCN Belgique 2017), which motivated the newly arising grassroots movement SYNAPARCAM to engage in a series of rallies. This has been connected with the transnational alliance of SOCFIN-affected communities, coordinated by ReAct (2015). At the time, a broad set of human rights, environmental, and social justice NGOs started challenging SOCFIN over its plantations, especially in Cameroon, Sierra Leone, and Cambodia. This includes Bread for All, FIAN, the International Federation for Human Rights (FIDH), Friends of the Earth, Greenpeace, and the Oakland Institute (Sändig et al. 2018)

In fact, SOCAPALM did install dialogue platforms in response to the activist challenges. At various points, the company invited community leaders and state actors for discussing issues. Yet, this has been flawed as the main grassroots’ movement SYNAPARCAM was rarely included and as the company showed no intention to change its practices. In the words of the leading activist Emmanuel Elong (Trait d’Union Magazine 2017, p. 32):

The communities of the said plantations are still demanding a real dialogue with SOCAPALM, because the meetings that this company organizes with the traditional chiefs and the administrative authorities are only empty shells where people come to talk without taking real resolutions and especially without ensuring a follow-up.

SOCFIN eventually expanded its CSR engagement in 2018. At SOCAPALM, the company employed the consultancy Earthworm (formerly The Forst Trust) to engage with local communities. More broadly, SOCFIN increased transparency on its business operations, started social responsibility reporting, and sought certification within the Roundtable on Sustainable Palm Oil (RSPO; see SOCFIN 2022). Yet, these CSR actions have not curbed contestation, as they are too little, too late. Key issues, most notably land control and repression, have not improved, and these half-hearted CSR efforts have met a highly mobilized activist network. The CSR endorsement even created new opportunities, as activists used company-provided information and the RSPO for the struggle.

SOMDIAA, in contrast, has been more open to community dialogue. SOSUCAM pursued ad-hoc consultation after the contentious episode of 2010 and created a trimestral meeting platform of company representatives, state officials, and local chiefs. Meeting minutes, job offers, and other company announcements were clearly displayed on information boards in the villages. Moreover, SOSUCAM’s CSR department regularly engaged with the *Comités Riverains de Veilles* (CRV). These community-based advocacy groups were created on the initiative of the CAVT, a faith-based NGO belonging to the Catholic Church and supported by MISEREOR (Germany). CRVs monitor the fulfillment of the company’s social and environmental pledges and seek to apply for micro-projects provided by the SOSUCAM foundation. The meetings between the CRVs and SOSUCAM alternated between the company offices and the villages for broader participation and transparency.

These dialogue opportunities, alongside occasional concessions by the company, demobilized potential protest. A leading figure of the 2010 protests recounted in the interview that they ended the contention in response to SOSUCAM’s CSR efforts: “So, we made peace and said we will work in partnership with the [SOSUCAM] foundation.”^15^ Other community leaders similarly expressed that they have access to the company and that it credibly addresses issues: “[N]evertheless, our requests occasionally succeed. The needs are heard at all times by the company.”^16^ In this context, a CRV representative explained that, in his view, there was no longer any need for contentious actions since the struggle is at the “next stage”, which comprises dialogue and concessions.^17^ But other interviewees were skeptical, questioning SOSUCAM’s intentions:

In my opinion, the CAVT is directed by the company. (...) They have done well to organize social platforms, to push us away from brutal actions. But when we were doing our actions, we got things from the company. But they have come to break our backs with their platforms. Now we are being bypassed, we are being bypassed. They say ‘tomorrow you’ll have a well and so on’, but in the end, we have got nothing.^18^

Hence, SOSUCAM effectively channeled local discontent and opposition from the CRVs into institutionalized dialogue platforms. Through occasional concessions, it set incentives for continuing dialogue rather than engaging in resistance. SOMDIAA used this approach more broadly at its plantations (Partner Africa 2017c, pp. 19–20). Crucially, the CSR actions were rather substantial at the time, as interviewees recognized the benefits that would accrue, and CSR was quickly endorsed in response to the 2010 contestation and the wider international attention on land grabbing.

Considering this demobilizing effect of CSR, SOSUCAM’s recent shift away from dialogue and conciliation led to increased contestation. The company refrained from renewing its social and environmental impact assessments and discontinued the dialogue with the CRV around 2018 (ReAct Transnational 2022, p. 58). This pushed the CRVs towards resistance. In November 2020, the CAVT (at the head of the CRVs) filed an NCP complaint in France (PCN France 2022). In mid-2021, SOMDIAA took the unprecedented step of withdrawing from the NCP process, refusing to negotiate at the French embassy rather than at the plantation site. Hence, by discontinuing core CSR practices, SOMDIAA has recently provoked renewed contestation.

### Opportunities and threats for transnational activism

Looking at the transnational level, the action opportunities are largely similar given the above-mentioned structural similarities of the selected cases. Some different corporate structures, however, help to explain why contestations unfolded differently. As the literature suggests, larger companies become more contested and INGOs prefer challenging companies from their own country (King 2008; Hatte and Koenig 2020). In these regards, SOCFIN’s larger size and multinational structure with headquarters in four countries contributed to the more extensive INGO involvement. SOMDIAA and the Castel Group, in contrast, remain relatively less known due to their smaller size, and they are mainly only relevant for French NGOs. The country link for activism is evident but not decisive, e.g. considering that a US-based investigate group also reported on SOMDIAA/Castel Group.

Besides these structural differences, corporate practices shaped the differing contestations. Contrary to previous research (Bartley and Child 2014; Graafland 2018), I do not find that CSR efforts by the parent companies per se increase activist challenges. Instead, SOMDIAA’s substantial CSR practices lowered contestation for many years, while SOCFIN’s shallow CSR actions have not reduced resistance. Specifically, I find two pathways, by which corporate practices at the plantation level also structure INGO involvement, and argue that SOCFIN’s repressive actions have amplified transnational contestation.

The cases show a gatekeeper logic for transnationalization. Domestic NGOs in the Global South, which have more information on local settings, can bring issues to international attention (Sändig et al. 2018). Interviews with Cameroonian NGOs revealed that some of them considered the practices of SOSUCAM more in line with CSR standards, whereas those of SOCAPALM were seen as more problematic. As one NGO staff member expressed:

The realities of the local communities is really deplorable, sad. No social responsibility on the part of SOCAPALM. (…) [SOSUCAM] is a little more open to the grievances of the people and tries to respect certain commitments. It is not really great, but there is a will on its part and some initiatives.^19^

For these domestic NGOs, there has been greater urgency to address issues at SOCAPALM and therefore to raise international attention on SOCFIN.

The different extent of local contestation, itself shaped through corporate practices on the ground, also impacts the engagement of INGOs. While the latter have various organizational and political reasons for their case selection, for smaller INGOs, in particular, already ongoing community mobilization and local resistance is a precondition for their involvement.^20^ Since SOSUCAM demobilized local contention for a long time through its CSR practices, it also limited INGO involvement. In contrast, the corporate practices of SOCAPALM and other SOCFIN branches have led to local resistance, especially against land grabbing and repression, which has made the case relevant to INGOs, too.

At the transnational level, SOCFIN undertook repressive actions, which threatened activists and journalists and which “backfired” by increasing contention. Since 2009, SOCFIN and especially the Bolloré Group filed at least 20 defamation suits against NGOs and media houses (including Le Monde, Le Point, and France Info, see RSF 2018). Accused NGOs and journalists denounce this as strategic lawsuits against public participation (SLAPPs). Such repression can have complex effects (Davenport et al. 2005): it can intimidate and constrain activists but also encourage further resistance—the latter occurred here. INGO interviewees described the court cases as costly and time consuming, but also as strengthening their resolve and fostering solidarity and activist networking.^21^ Most importantly, the lawsuits and media reporting greatly increased the public attention on Vincent Bolloré and SOCFIN. This attracted further INGOs to the struggle, which then also created new support and action opportunities for local activist networks in Cameroon, Sierra Leone, and beyond.

To some extent, this escalatory process is endogenous to the resistance, amplifying the struggle. SOCFIN’s recent attempts to use CSR for lowering contention have been unsuccessful. INGOs see this as mere “greenwashing”, arguing that the exploitative business practices and repression have continued (ReAct 2019). In contrast, SOMDIAA and the Castel Group have evaded such escalatory spirals. The leading figures have taken much lower profile than Vincent Bolloré. SOMDIAA’s long-standing CEO Alexandre Vilgrain was known mainly in the French business community, and Pierre Castel (founder and CEO of his family empire) remains largely out of the public eye. They have not responded to INGO activism with repression, but they have also faced less INGO scrutiny from the outset. Conducting research and campaigning on SOMDIAA and the Castel Group are therefore still higher, which discourages especially smaller INGOs.^22^ They have incentives to bandwagon more on established cases such as SOCFIN.

## Conclusion

The paper started from the observation that research on contention against large-scale agricultural investment so far has focused mainly on community characteristics, political and activist support networks, the broader political context, and framing processes (Gagné 2019; Rutten et al. 2017; Prause 2019). In doing so, the role of the investing companies, and particularly of CSR practices, has been surprisingly neglected. To address this, I integrated insights from research on mining and other investment conflicts on the CSR-contention nexus into the opportunities and threats framework of social movement studies. Regarding two major European-run plantation companies from Cameroon, which have been differently contested, I examined how corporate practices shaped the grievances, threats, and opportunities that underlie contentious actions, both on the ground and transnationally.

Overall, I find a demobilizing effect of timely and substantial CSR practices, whereas non-substantial and belated CSR efforts and, most importantly, corporate actions that violate CSR tenets provoke resistance. SOCAPALM and SOCFIN have made numerous rhetorical commitments to responsible investment, but actual practices have lagged behind. Their practices of land acquisition, non-dialogue, limited development contributions, and repression have provoked resistance. Although SOCFIN expanded its CSR efforts in 2018, this has not demobilized the local-transnational struggle. Its CSR practices remain non-substantial, as land conflicts and repression of critics persist, and they come too late, as the SOCFIN challengers are highly mobilized.

SOSUCAM and SOMDIAA, in contrast, long demobilized contestation through CSR practices. Around 2011, SOSUCAM started to engage in dialogue with local communities, respect local land needs, and provide social projects for compensation of its plantation expansion. This gave local leaders and activists an opportunity for raising claims through institutionalized dialogue platforms rather than contentious actions. Yet, when the company discontinued most of these CSR efforts around 2019, new grievances and renewed contention arose, which also attracted INGOs.

Thus, I show that some previous findings on CSR effects, especially from mining conflict research, apply to contention over agricultural investment, too. My key finding on the demobilizing effects of substantial CSR efforts reflects Haslam’s argument that CSR increases the costs of protest, when it effectively reduces local perceptions of risk and uncertainty, channels public participation into dialogue processes, and distributes significant material benefits (Haslam 2021). Vice versa, non-substantial, belated, and discontinued CSR practices provoke (or fail to curb) resistance, both locally and transnationally. Hence, contrary to previous research, I do not find that CSR increases contention by creating new grievances, distributional conflicts, and struggles over participation (Anguelovski 2011; Bezzola 2022). My observations, furthermore, contradict the argument that CSR endorsement increases contestation at the headquarters’ level (Bartley and Child 2014; Graafland 2018). Rather, in the examined cases, substantial and timely CSR practices also undercut transnational contestation.

The different extent of contestation, however, also partly results from different corporate structural properties as well as endogenous escalation dynamics. SOCFIN’s somewhat larger size, multiple headquarters, and faster pace of plantation development made resistance more probable in this case. This underlines research on the relevance of company size and headquarter location for transnational contention (Bartley and Child 2014; Hatte and Koenig 2020). Moreover, there have been self-reinforcing escalation processes at play in the SOCFIN case, on the ground over everyday resistance and transnationally from “backfiring” repression.

The paper provides further insights for theories on resistance against large-scale investment projects. It corroborates previous research on the relevance of NGOs for these struggles (Gerber 2011; Temper 2019) but also addresses the neglected question of how local struggles become transnationalized. Here, I show that domestic NGOs from the Global South act as gatekeepers and that local mobilization is a precondition for involvement, especially of smaller INGOs (Sändig et al. 2020). The paper also illustrates the under-researched issue of repression against transnational activists. While studies so far have focused mostly on repression at the investment site (Dell’Angelo et al. 2021), the SOCFIN case shows important escalatory effects of the repression at the company’s headquarters.

As final note of caution on CSR, local communities certainly tend to be better off if companies pursue substantial CSR practices than if they do not. The large-scale plantation model, nevertheless, remains fundamentally flawed and exploitative. Furthering smallholder agriculture and agroecology promises more socially and ecologically sustainable futures (McKeon 2015).

## Footnotes

1 In this article I use the terms contention, contentious actions, contestation, protest, and resistance synonymously to denote public actions in which people collectively raise claims towards companies (and partly also state actors).

2 Guidance taken from the Principles for Responsible Investment in Agriculture and Food Systems (PRAI), endorsed within the UN Food and Agriculture Organization (FAO, see 2014).

3 Information on these companies was mainly collected from their websites, CSR reports (including SOCAPALM (2018); SOMDIAA 2017), and NGO reports (e.g. SNJP 2016).

4 In 2023, they sought to take full control by acquiring the remaining shares.

5 Interviews were conducted at the SOCAPALM sites Dibombari, Mbongo/Mbambou, and Edéa, and interviews for SOSUCAM at Mbandjock, Nkoteng, and surrounding villages. For the sensitivity of the topic, names of interviewees and organizations are generally withheld. Interview quotes were translated from French by using www.deepl.com.

6 An important constraint for the data collection was also that, for different reasons, a planned second field research trip could not take place.

7 Interview with two villagers, nearby Nkoteng, 5 December 2017.

8 Interview with young man, Dibombari site, 29 November 2017.

9 Interview with a villager, Dibombari site, 28 November 2017.

10 Interview with local activist, Mbongo site, 2 December 2017.

11 Interview with local activist, Dibombari site, 1 December 2017.

12 Interview with local activist, Yaoundé, 12 December 2017.

13 Yet, this also divided the local communities and created incentives for contention because those displaced for the initial plantation now claim a similar lease share.

14 Interview with a group of women, nearby Nkoteng, 5 December 2017.

15 Interview with a local activist, Yaoundé, 11 December 2017.

16 Interview with a community leader, nearby Nkoteng, 6 December 2017.

17 Interview with a local activist, Yaoundé, 12 December 2017.

18 Interview with two CRV members, nearby Nkoteng, 8 December 2017.

19 Interview with NGO staff member, Yaoundé, 22 November 2017.

20 Interviews with INGO staff, online, 24 April and 8 May 2023.

21 Interviews with INGO staff, online, 24 April, 8 May, 15 May 2023.

22 Interviews with NGO staff members at Yaoundé (13 December 2017) and online (24 April 2023).
